# Sympathetic activation and heart rate thresholds for cardiovascular risk in chronic kidney disease

**DOI:** 10.1097/HJH.0000000000003179

**Published:** 2022-07-05

**Authors:** Guido Grassi, Bianca Fowler, Beatrice Scali, Federica Rossi, Elena Motto, Federico Pieruzzi, Giuseppe Mancia

**Affiliations:** a Clinica Medica; b Clinica Nefrologica, Department of Medicine and Surgery, University Milano-Bicocca; c University Milano-Bicocca, Milan, Italy

**Keywords:** cardiovascular morbidity, cardiovascular mortality, chronic kidney disease, heart rate, plasma norepinephrine, sympathetic activity, sympathetic nerve traffic

## Abstract

**Aim:**

The current study was designed at assessing whether the sympathetic cardiovascular drive (SNS) is differently activated in chronic kidney disease (CKD) patients displaying less or more elevated resting heart rate (HR) values. It was also designed at determining at which HR cutoff value the SNS displays a greater activation.

**Methods:**

In 95 CKD middle-age patients we evaluated muscle sympathetic nerve activity (MSNA, microneurography) and venous plasma norepinephrine (HPLC assay), subdividing the patients in different groups according to their resting clinic and 24-h HR.

**Results:**

In CKD progressively greater values of clinic or 24-h HR were associated with a progressive increase in both MSNA and norepinephrine. HR cutoff values indicated by large-scale clinical trials for determining cardiorenal risk, that is more than 80 bpm, were associated with MSNA values significantly greater than the ones detected in patients with lower HR, this being the case also for norepinephrine. Both MSNA and norepinephrine were significantly related to clinic (*r* = 0.47, *P* < 0.0001 and *r* = 0.26, *P* < 0.0001, respectively) and 24-h (*r* = 0.42, *P* < 0.0001 and *r* = 0.27, *P* < 0.0001, respectively) HR. MSNA, norepinephrine, but not HR, were significantly and inversely related to estimated glomerular filtration rate (eGFR) values (*r* = −0.47, *r* = −0.23, *P* < 0.0001 and *P* < 0.02, respectively).

**Conclusion:**

In CKD both clinic and 24-h HR values greater than 80 bpm are associated with an enhanced sympathetic activation, which parallelles for magnitude the HR elevations. The sensitivity of HR as sympathetic marker is limited; however, no significant relationship being detected between HR and eGFR or left ventricular mass index.

Studies based on the evaluation of indirect and direct markers of neuroadrenergic cardiovascular drive have unequivocally shown that chronic kidney disease (CKD) is characterized by a marked sympathetic activation [1–10], whose detection is common not only in the advanced but also in the earlier stages of this clinical condition [2,3,11]. Evidence has been also obtained that the sympathetic abnormalities become progressively more pronounced as renal function progressively declines [12], predicting, independently on other confounders, the development of fatal and nonfatal cardiovascular and renal outcomes [13].

The above mentioned conclusions have been based on the results of the studies which evaluated sympathetic cardiovascular function assessing circulating venous plasma levels of the adrenergic neurotransmitter norepinephrine [1,3,13], performing measurement of the net release of norepinephrine secretion from sympathetic nerve terminals [14], analyzing power spectral oscillations of heart rate (HR) in the high-frequency bands [4,5] and directly recording sympathetic nerve traffic in peripheral nerves via the microneurographic technique [6–10,11,12]. Recently, however, the information gained on this issue has been implemented with the finding that in CKD even a hemodynamic variable of very easy measurement, such as clinic HR, may reflect, although with some limitations, sympathetic cardiovascular drive (SNS) [15]. Evidence has been also obtained that HR may represent a marker of cardiorenal risk, being significantly associated with renal outcomes and disease progression, as reported in three major clinical trials, that is, the ONgoing Telmisartan Alone and in combination with Ramipril Global End-point Trial [16], the Telmisartan Randomized AssessmeNt Study in ACE iNtolerant subjects with cardiovascular Disease [16] and the Multiple Intervention and Audit in Renal Disease to Optimize care study [17]. However, no information is available so far on whether the sympathetic nervous system is differently activated in CKD patients displaying less or more elevated resting HR values. This information is essential to determine whether more or less elevated HR values are capable to reflect in this disease a different degree of sympathetic activation.

The current study was designed to address this issue by assessing sympathetic drive in a large sample of CKD patients displaying different resting levels of HR. Two were the unique features of the study. First, the evaluation of the sympathetic function was based on two different independent neuroadrenergic markers, namely venous plasma norepinephrine and efferent postganglionic sympathetic nerve activity (MSNA) directly recorded via the microneurographic technique in the peroneal nerve. Second, the assessment of HR included not only clinic but also 24-h values, allowing to relate static and dynamic figures of this hemodynamic variable to the above mentioned sympathetic markers.

## METHODS

### Population

The study population consisted of 95 patients of both sexes (76 males and 19 females) with an age range between 40 and 72 years. For all patients the evaluation was done retrospectively and it was based on the detection of an HR value below or above 70 bpm at the office visit performed the day preceding the microneurographic nerve traffic recording session made in the frame of different investigations carried out between 2015 and 2021. All individuals included in the study were in sinus rhythm, and no subject had a history of myocardial infarction in the 12 months before the study or clinical or laboratory evidence of valvular heart disease, congestive heart failure, thyroid dysfunction, diabetes mellitus, metabolic syndrome, obesity, or any other condition known to affect autonomic modulation of the cardiovascular system [18]. Recruited patients were affected by CKD of different clinical severity according to the estimated glomerular filtration rate (eGFR) values, that is eGFR at least 90 ml/min/1.73 m^2^, eGFR between 89 and 60 ml/min/1.73 m^2^, eGFR between 59 and 45 ml/min/1.73 m^2^ (corresponding to CKD stage 3A), between 44 and 30 ml/min/1.73 m^2^ (corresponding to stage 3B) and end-stage CKD patients maintained on chronic hemodialytic treatment [19]. Patients were recruited if they displayed a stable clinical state in the 3 months prior to their inclusion in the study. The majority of the patients displayed a sedentary behavior, particularly in the groups characterized by a more advanced CKD. They were under antihypertensive drug treatment with angiotensin converting enzyme inhibitors, angiotensin II receptor antagonists, loop diuretics, and calcium antagonists. Beta-blocking drugs, if present, were withdrawn 1 week prior to the study. Patients were evaluated on an outpatient basis and gave their written consent to the study after being informed of its nature and purpose. The study protocol was approved by the Ethics Committee of one of the institutions involved.

### Measurements

Measurements included BMI, sphygmomanometric and beat-to-beat finger SBP and DBP via a validated instrument (Ohmeda 2003; Finapres, Englewood, Florida, USA) [11,15], HR (EKG) and respiration rate (pneumotacograph). They also included MSNA via the microneurographic technique [3,11,15,18], venous plasma norepinephrine via high-performance liquid chromatography with electrochemical detection (Machery-Nagel ET 200/4 Nucleosil 100-5 C18 column, Machery-Nagel, and Waters 460 electrochemical detector; Waters GmbH, Eschborn, Germany) [20], and an echocardiographic assessment of the end-diastolic and end-systolic left ventricular (LV) internal diameters, interventricular septum thickness, and posterior wall thickness, left atrial diameters and LV ejection fraction, measured from the four-chamber apical projection using the product area times length [21,22]. Echocardiographic data also included mitral flow [early diastolic peak flow velocity (E wave) and late diastolic peak flow velocity (A wave)] and flow at the LV outflow tract values. LV mass index (LVMI) was calculated by the Devereux formula and normalized to body surface area [22]. An EKG-Holter monitoring was performed during the 24-h period in the days preceding the evaluation of the sympathetic neural function.

Simultaneous MSNA, beat-to-beat HR, and blood pressure (BP) recordings were digitized with a sampling frequency of 1000 Hz (PowerLab Recording System Model ML870 8/30; AD Instruments, Bella Vista, New South Wales, Australia). MSNA was quantified over a 30-min period as bursts incidence over time (bursts/min) [11,17,23]. This quantification has been shown to be highly reproducible, that is to differ by only 4.3% when assessed on two separate occasions [23].

### Protocol and data analysis

All participants were examined in the morning after a light breakfast and an overnight abstinence from alcohol and coffee consumption. They were asked to assume the supine position, after which three sphygmomanometric BP and HR (palpatory method, radial artery) were obtained. Following the BP and HR measurements, the patients were fitted with an intravenous cannula and the devices to measure finger BP and to record an EKG. Blood samples were taken 30 min after positioning the venous cannula. A microelectrode was then inserted into a peroneal nerve to obtain MSNA, which was recorded together with finger BP and the EKG during a 30-min period. Data were collected in a semidark and quiet room kept at a constant temperature of 20–22°C. As mentioned above, the study had a retrospective nature and included data collected and already blindly analyzed for other microneurographic studies not related to the objective of the present investigation. This allowed to avoid any potential bias in data analysis. Values from individual participants were averaged (see below) and expressed as means ± SEM. The study population was subdivided into three different groups according to the clinic HR values displayed at rest by the patients. Comparisons between groups were made by two-way analysis of variance, using the Student *t* test for unpaired observations or by chi-square statistic to determine their differences. The Pearson correlation coefficient was used to determine the relationships between HR, MSNA, norepinephrine and other parameters, a *P* less than 0.05 being taken as the minimal level of statistical significance. All statistical analyses were performed by SAS software version 9.4 (SAS Institute Inc, Cary, North Carolina, USA).

## RESULTS

As shown in Table 1 the three groups of CKD patients characterized by resting clinic HR values below 70, between 70 and 79, and above 80 bpm displayed similar sex distribution and superimposable age. No significant difference between groups was found as far as BMI, SBP and DBP, serum hemoglobin, serum glucose, plasma creatinine are concerned. Left ventricular ejection fraction was similar in the three groups, which also showed similar *E*/*A* values. LVMI was greater, although non significantly, in the group of patients showing HR values more than 80 than in the other two groups. EGFR was similarly impaired in the three groups, although there was a tendency of CKD stages to be more advanced in the group with HR values more than 80 than in the other two. A similar trend was observed for the percentage of CKD patients with a history of hypertension.

**TABLE 1:** Demographic, hemodynamic, and clinical characteristics of patients with chronic kidney disease classified in three groups accordingly to different clinic heart rate values

Variable	HR < 70 bpm, *n* = 29	HR 70–79 bpm, *n* = 41	HR > 80 bpm, *n* = 25
Age (years)	58.8 ± 2.2	59.6 ± 1.4	60.8 ± 2.5
Sex (M/F, *n*)	22/7	36/5	19/6
BMI (kg/m^2^)	26.7 ± 0.7	26.3 ± 0.4	25.7 ± 0.9
Clinic SBP (mmHg)	142.8 ± 3.1	143.4 ± 2.3	145.5 ± 2.9
Clinic DBP (mmHg)	77.1 ± 2.6	78.9 ± 22	79.6 ± 2.7
LVEF (%)	59.0 ± 1.3	58.4 ± 0.8	57.7 ± 1.5
*E*/*A* ratio (a.u.)	1.11 ± 0.2	1.12 ± 0.1	1.09 ± 0.2
LVMI (g/m^2^)	113.0 ± 2.2	111.7 ± 1.5	117.2 ± 2.5
Hemoglobin (g/dl)	13.1 ± 0.4	13.3 ± 0.2	12.9 ± 0.0.3
Serum glucose (mg/dl)	88.2 ± 4.6	87.7 ± 4.2	88.9 ± 94.7
eGFR (ml/min 1.73 m^2^)	57.3 ± 1.4	58.7 ± 1.2	56.4 ± 1.3
Respiration rate (bpm)	16.9 ± 1.1	17.4 ± 0.9	16.6 ± 1.3
CKD stage (%)			
1	17.2	26.8	4.0
2	24.1	26.8	8.0
3A	24.1	19.5	32.0
3B	27.7	17.8	44.0
HD	6.9	9.1	12.0
Hypertension (%)	76.1	79.6	86.9
No. of drugs/day	3.2	3.1	3.7

Data are shown as means SEM. a.u., arbitrary units; CKD, chronic kidney disease; eGFR, estimated glomerular filtration rate; F, females; HD, hemodialysis; LVEF, left ventricular ejection fraction; LVMI, left ventricular mass index; M, males.

**FIGURE 1:**
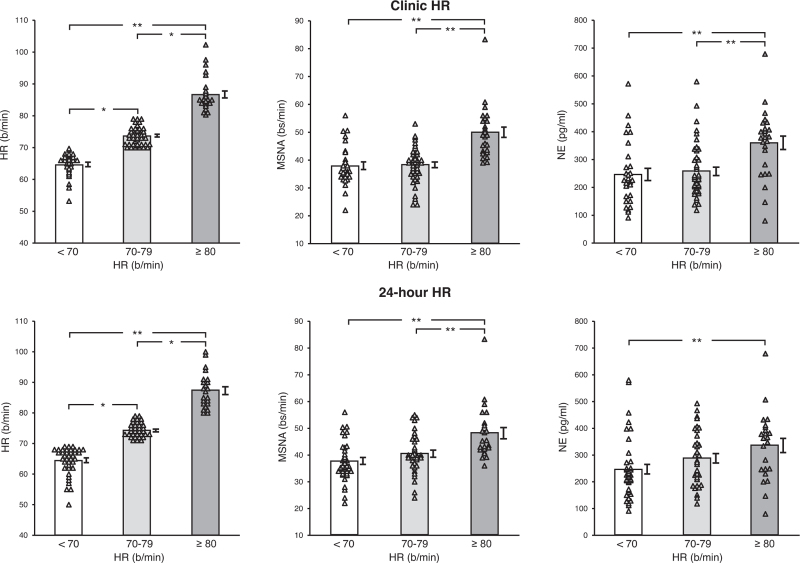
*Upper panels:* Individual and average values (±SEM) of clinic heart rate, muscle sympathetic nerve activity, and venous plasma norepinephrine in the groups of patients with chronic renal failure with resting clinic heart rate less than 70 bpm, between 70 and 79 bpm, and more than 80 bpm. *Lower panels:* Individual and average values (±SEM) of 24-h heart rate, muscle sympathetic nerve activity, and venous plasma norepinephrine in the groups of patients with chronic renal failure with resting 24-h heart rate less than 70 bpm, between 70 and 79 bpm, and more than 80 bpm. Bs/min indicates bursts/minute. Asterisks (^∗^*P* < 0.05, ^∗∗^*P* < 0.01) refer to the statistical significance between groups.

Individual and average clinic HR values assessed in the three CKD groups are shown in Fig. 1, which also displays individual and average data obtained for 24-h HR monitoring, as well as the corresponding individual and average MSNA and venous plasma norepinephrine values. As expected, in CKD patients resting HR showed values progressively and significantly greater from the group with an HR below 70 bpm to the ones displaying HR between 70 and 79 and above 80 bpm (Fig. 1, left upper panel). This was the case also for 24-h HR values (Fig. 1, left lower panel). More importantly, although consistent interindividual differences were found, patients with clinic HR values greater than 80 bpm displayed MSNA and norepinephrine values significantly increased when compared with those found in CKD with HR between 70 and 79 bpm or below 70 bpm (Fig. 1, upper middle and right panels). A similar behavior was observed when the 24-h HR data were examined (Fig. 1, lower middle and right panels).

**FIGURE 2:**
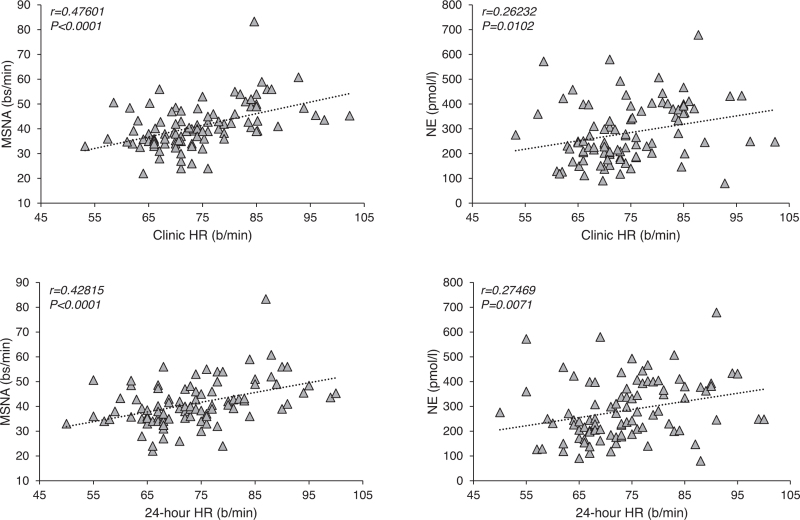
*Upper panels:* Regressing clinic heart rate on muscle sympathetic nerve activity, expressed as bursts frequency over time (bursts per minute, bs/min, left) and plasma norepinephrine (right) in 95 patients with chronic renal failure. Correlation coefficients (*r*) and *P* values are shown. *Lower panels:* Regressing 24-h heart rate on muscle sympathetic nerve activity , expressed as bursts frequency over time (bursts per minute, left) and venous plasma norepinephrine (left) in the 95 patients with chronic renal failure. Correlation coefficients (*r*) and *P* values are shown.

In the group as a whole both MSNA and plasma norepinephrine showed highly significant direct relationships with clinic HR, the correlation being closer for MSNA than for norepinephrine (Fig. 2, upper panels). Similar significant relationships were found between 24-h HR values and MSNA or norepinephrine (Fig. 2, lower panels). However, when the same relationships were sought separately in the three different groups of CKD patients no significant correlation was found (data not shown).

**FIGURE 3:**
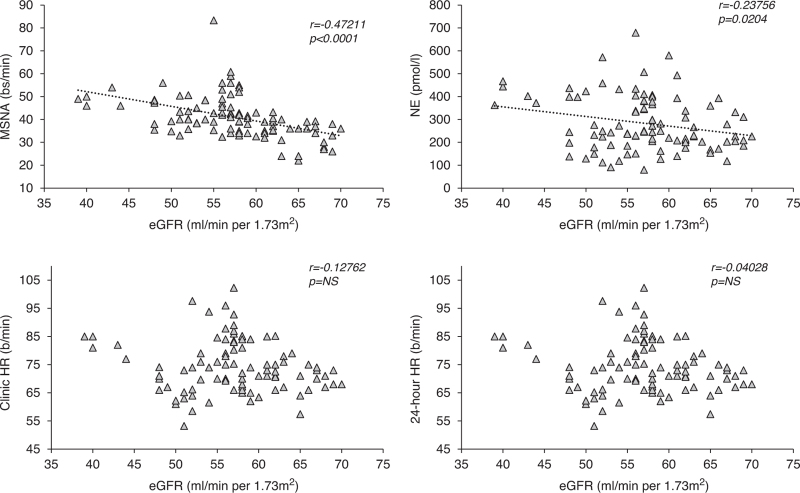
*Upper panels:* Regressing muscle sympathetic nerve activity, expressed as bursts frequency over time (bursts per minute, bs/min, left), on values of the estimated glomerular filtration rate and plasma norepinephrine (right) in 95 patients with chronic renal failure. Correlation coefficients (*r*) and *P* values are shown. *Lower panels:* Regressing clinic heart rate (left panel) on values of the estimated glomerular filtration rate and 24-h heart rate (right) in 95 patients with chronic renal failure. Correlation coefficients (*r*) and *P* values are shown.

As shown in Fig. 3, upper panels, both MSNA and norepinephrine displayed a significant inverse relationship with eGFR. This was not the case, however, for clinic HR and for 24-h HR (Fig. 3, lower panels). In addition, while both MSNA and norepinephrine were directly and significantly related to LVMI (*r* = 0.34 and *r* = 0.47, respectively, *P* *<* 0.001 for both), no significant correlation was detected for clinic and 24-h HR (*r* = 0.12 and *r* = 0.18, *P* = NS for both).

## DISCUSSION

Three are the major novel findings of the current study. The first one concerns the evidence that in CKD patients clinic HR values above 80 bpm are accompanied by an activation of the sympathetic nervous system significantly and markedly greater for magnitude than the one detected in CKD patients displaying clinic HR values below 70 or between 70 and 79 bpm. The second new finding refers to the evidence that the above mentioned 80 bpm HR cutoff value applies not only to clinic but also to 24-h HR. A further new finding refers to the fact that the relationships with HR were noticeable not only when sympathetic function was evaluated by the sophisticated approach based on direct recording of MSNA but also by venous plasma norepinephrine assay. In the former case, however, the strength of the correlation was more pronounced than in the case of norepinephrine. This confirms one more time the greater sensitivity of the technique based on microneurographic recording of sympathetic nerve traffic compared with the biochemical assay of the adrenergic neurotransmitter already reported in previous studies [12,15,18,23,24]. Taken together these findings strongly support the choice of HR cutoff amounting to 80 bpm for identifying CKD patients with a pronounced sympathetic activation. In this context, it should be emphasized that the above mentioned clinic HR cutoff value was superimposable to the one seen in essential hypertensive patients [25]. It was greater for magnitude, however, than the cutoff value detected in patients with congestive heart failure, which was positioned at a HR value amounting to 70 bpm [26]. It is likely that these differences may depend on the fact that because the neuroadrenergic activation is greater for magnitude in chronic heart failure than in hypertension or in CKD [18,23], the sympathetic alteration can be more easily detected at lower HR values.

Other additional results of our study are worthy to mention. In the group of CKD patients as a whole we found that both clinic and 24-h HR values were significantly related to the other two indices of sympathetic tone employed in the current study, namely MSNA and plasma norepinephrine. This finding may indicate the possible use of HR as ‘surrogate’ marker of sympathetic drive when, such it usually happens in current clinical practice, other more sensitive adrenergic indices are not available. It should be emphasized, however, that the sensitivity of the approach based on HR remains lower than the other two, considering that no correlation between HR and the other sympathetic indices achieved the minimal level of statistical significance when the analysis was restricted to each single subgroup of CKD patients characterized by progressively greater HR values. The limited sensitivity of the approach based on measurement of HR is further supported by the evidence that this variable, both when assessed in the clinical setting by the palpatory method of the radial artery and when obtained by 24-h monitoring, fails to show any significant relation with the eGFR values. Similarly no significant correlation was detected between clinic and 24-h HR and the increased values of LVMI detected in CKD, known to be under sympathetic influences [27,28]. This is supported in the current study by the significant correlation found between MSNA and norepinephrine and echocardiographic measures of LV mass. Finally it should be emphasized that about three quarters of CKD patients with HR greater than 80 bpm displayed a stage 3 of the disease at variance from what found in the other two groups of patients in which stage 3 CKD was detected in a much less elevated number of patients. This suggests that the more advanced CKD state may be responsible for the augmented SNS activation and the greater levels of HR.

Our study has limitations but also clinical implications. One limitation concerns the fact that our study had a retrospective nature. This approach, however, allowed us to analyze one of the largest database of microneurographic indices of sympathetic drive in patients with CKD. A further limitation was related to the fact that we evaluated CKD patients under standard drug treatment potentially interfering with the sympathetic drive. However, mention should be done that beta-blockers, that is, drugs directly acting on HR [29], were withdrawn 5–7 days prior to the study. In addition the drugs used for the treatment of CKD have been shown to exert effects on the sympathetic nervous system which counterbalance each other, some drugs enhancing while others inhibiting cardiovascular sympathetic neural drive [18,29]. On average this would finally result in a neutral sympathetic effect.

The clinical implication refers to the evidence that at a resting clinic HR value more than 80 bpm neuroadrenergic cardiovascular drive is markedly activated, strongly supporting the European Society of Cardiology/European Society of Hypertension guidelines to utilize the above mentioned HR value for defining cardiovascular risk profile in hypertensive patients with CKD [30].

## ACKNOWLEDGEMENTS

### Conflicts of interest

There are no conflicts of interest.
